# Defective but promising: evaluating the utility of currently available bioinformatic pipelines for detecting defective viral genomes in RNA-Seq data

**DOI:** 10.1099/jgv.0.002176

**Published:** 2025-11-17

**Authors:** Anthony Taylor, Cristina Rosa, Marco Archetti

**Affiliations:** 1 The Huck Institutes for the Life Sciences Intercollege Program of Plant Biology, The Pennsylvania State University, Pennsylvania 16803, PA, USA; 2 Department of Plant Pathology and Environmental Microbiology, College of Agricultural Arts and Sciences, The Pennsylvania State University, Pennsylvania 16803, PA, USA; 3 Department of Biology, Eberly College of Science, The Pennsylvania State University, Pennsylvania 16803, PA, USA

**Keywords:** bioinformatics, defective interfering RNA, defective interfering virus, plant virus

## Abstract

Defective viral genomes (DVGs) affect viral dynamics, pathogenicity and evolution, have been found in many *in vivo* viral infections, and in theory can be detected from sequencing data. We explored the utility of the currently available bioinformatic programs ViReMa, DI-tector, DVGfinder, DG-Seq and VODKA2 for identifying junction points in plant virus high-throughput sequencing data, looking at whether the outputs from these bioinformatic tools generally agree and exploring the possibility of using these tools to help us understand whether DVGs are consistently generated and maintained in a specific virus-host combination. We conducted a meta-analysis of eight previously published RNA sequencing datasets utilizing all five programs and compared the degree of output overlap, the most common junctions present in each output and whether these junctions match previously reported junctions for that virus. Our results demonstrate a low degree of agreement regarding identified junctions between programs, including the most frequently identified one, although the most frequently identified junctions typically corresponded to large, disruptive deletions. We found preliminary support for our prevalence hypothesis, although we ultimately conclude that a more robust dataset generated expressly for testing this hypothesis will be required for a convincing answer. Finally, we suggest that when using bioinformatic programs to search for DVGs, it is best to run the same dataset through multiple programs and look at the overlap to inform decisions on downstream characterization.

**Impact Statement**

General interest in exploring viral quasispecies diversity through high-throughput sequencing is increasing, and several bioinformatic pipelines are available to search through HTS data for defective viral genomes (DVGs). We conducted a meta-analysis exploring the utility of the currently available bioinformatic programs in DVG identification, comparing whether the outputs from these tools generally agree. Our main result demonstrates a low degree of agreement in program output, suggesting that although bioinformatic workflows have a place in the toolbox of DVG research, multiple programs (along with other avenues of evidence) should be used on a dataset to better inform data analysis and interpretation.

## Data Summary

External software used

1. https://www.sourceforge.net/projects/virema (ViReMa v0.29)
2. http://www.di-tector.cyame.eu/(DI-tector v0.6)
3. https://rnajournal.cshlp.org/content/26/12/1905/suppl/DC1 (DG-seq)
4. https://github.com/MJmaolu/DVGfinder (DVGfinder v2)
5. https://github.com/lopezlab-washu/VODKA2 (VODKA2 (2.0))

External datasets used (from NCBI SRA, information also in methods Table 1)

1. SRR6374495
2. SRR6374496
3. SRR10119525
4. SRR039711
5. SRR5571020
6. SRR5568966
7. SRR11928841
8. SRR10490212
9. SRR12953073
10. SRR35289193
11. SRR35289192
12. Synthetic dataset from [1]

Code repository: https://github.com/skybird99-anthony-taylor/DIs_in_RNAseq_data.

The authors confirm that all supporting data, code and protocols have been provided within the article or through supplementary data files.

## Introduction

### Defective viral genomes and how they can be found

Viruses exist in their hosts as a ‘quasispecies’, a population of closely related viral variants that arise by mutation during replication. The interactions between these variants influence the overall fitness, pathogenesis and evolution of the virus; hence, it is important to characterize all variants present in a viral infection. Defective viral genomes (DVGs) are incomplete viral genomes present in the quasispecies which can persist because of complementation in trans by other viral quasispecies members. These DVGs may play interesting roles in the progression of viral infection and the evolution of the viral quasispecies, from stimulation of innate immunity in animals and plants [2,3] to influencing the evolution of multipartism [4]. A subset of these defective genomes that outwardly interfere with the replication of other quasispecies members has aroused much interest, for they are known to lead to amelioration of symptoms, lower viral titre or drive the differential accumulation of viral proteins [5,7]. Genomes that fall into this category are called defective interfering (DI) genomes, viruses, RNAs, particles or molecules. They have been known to occur in animal virus quasispecies since the 1950s and are commonly found *in vitro* after multiple passages [7]. It is debated, however, whether DIs exist in ‘natural’ (non-passaged) infections under non-laboratory conditions or if only laboratory passaging generates the required m.o.i. and favourable conditions for their accumulation [8,11]. Laboratory work has concluded that at least for *Tombusvirus lycopersici* [commonly tomato bushy stunt virus (TBSV)], DIs do not require passaging to arise and DIs with a conserved genome organization will arise even in infections started with infectious clones [11]. In addition, field work has been published reporting DVGs in naturally infected plants [12,16], with DIs only isolated and characterized after multiple passages. However, one field study, specifically looking for DIs and/or other DVGs in field infections of TBSV, found nothing but a satellite and concluded that DVGs are not found naturally or exist below the limit of Northern blots’ detection [9].

Determining the natural range of DVGs, especially DIs, in viral infections is important for a robust understanding of viral ecology, for DVG-induced differences in disease severity likely influence virus–vector–host interactions [15,17, 18], and DIs have been considered for novel antiviral treatments [2,19,24]. Therefore, methods to quickly identify DVGs would be useful to multiple areas of virology. Although DIs are the point of interest when exploring the defective portion of the quasispecies, most identification techniques do not provide information on interfering capability. Therefore, for the remainder of this paper we will be referring to all defective members of the quasispecies as ‘DVGs’. DVGs can be detected by Northern blotting [25,30]; however, although reliable, Northern blotting has some disadvantages, including requiring probe optimization, needing to know at least part of the sequence of the virus and the need for a relatively high amount of RNA for detection. Reverse Transcription quantitative Polymerase Chain Reaction (RT-qPCR) can detect smaller amounts of RNA but also relies on prior knowledge of the sequence and primer optimization. Furthermore, it is difficult to use RT-qPCR or Northern blotting to identify DVGs with large deletions, since the deleted portion may include the sequences of the primers and probes, as well as DVGs that are a mosaic of small indels, because primers and probes may not bind well enough for proper hybridization or amplification.

In response to these drawbacks, a couple of recent papers have suggested that the increase in high-throughput sequencing data, along with bioinformatic pipelines to process it, will aid in the identification of DVGs [8,31]. As sequencing becomes cheaper and computational resources more sophisticated, the number of virome datasets accessible in public online repositories have increased, making bioinformatic methods of detecting DVGs more attractive. There are already several bioinformatic tools available that identify DVGs from high-throughput sequencing data: ViReMa [32]. DI-tector [33], DG-seq [34], DVGfinder [1], DVG-profiler [35] and VODKA, now replaced with VODKA2 [36]. A review of their function is described below.

### Bioinformatic pipelines for the identification of DVGs

The oldest program in the literature is Viral Recombination Mapper (ViReMa), which was published in 2014 [32] and is still receiving annual updates. It is built for detecting recombination junction sites, doing so by aligning the 5′ end of a read to a reference genome with a bowtie seed-based alignment specified by the user (default is 25 nt). It then makes a new segment by either extracting any unaligned nucleotides at the 3′ end of the read or by trimming the first nt from the read. This is done repeatedly until the whole read is either mapped or trimmed. If the whole read is trimmed, it is considered truly unaligned. If the read maps in multiple locations, these locations are noted in output files. The program outputs .txt files with microdeletions, deletions, insertions, substitutions and recombinations and has settings allowing the user to specify the length of the bowtie seed used as well as the minimum number of nucleotides that need to be missing to count the read as a recombination junction site. It is designed for short-read data but can be used with long-read datasets if Burrows–Wheeler alignment (bwa) is specified instead of bowtie in the argument --aligner bwa [32].

The second oldest program is DI-tector, which was published in 2018 and uses bwa to align data against host and virus reference genomes before discarding aligned reads and proceeding with unaligned and clipped reads. It then segments each unmapped read into two pieces: ‘first’ and ‘last,’ to map reads that contain junctions. For each read, the first segment’s size will vary from *X* to *N-X* and the last seg from *N-X* to *X* where *N* is the initial length of the read and *X* is the minimum segment length defined by the user with the argument --Min_Segment (default=20). The segmented reads are then aligned to the viral genome of interest using bwa-aln and bwa-samse. Reads for which the first and/or last segments are unmapped or multi-mapped are discarded, and mapped read segments (defined by their FLAG value: a base-ten number used to represent a binary number that corresponds to a true/false statement pertaining to the read. For example: a FLAG value of 4 means the read was not aligned) are processed and compared to cluster the ‘DVGs’ by similar junction location. If two reads map to different reference strands, they are labelled as copybacks. If two reads map to the same reference strand, their positions will be compared to characterize the deletion/insertion forms of the DVG [33].

Next is the DVG-profiler, published in 2019 as part of the High-performance Integrated Virtual Environment (HIVE) platform at the US Food and Drug Administration. It is not an aligner software and does not accept raw sequencing data, instead accepting pre-aligned and sorted short reads. It counts the number of alignments for each read, and, if it has more than one alignment and the alignments do not have a perfect score based on its scoring algorithm, it inspects the reads in pairs and finds the best-scored pair of alignments that corresponds to a junction. Like DI-tector, it clusters ‘DVGs’ by general location of the deletion, doing so via a peak-detection algorithm, where the breadth of what’s included in the cluster is defined by the user [35]. Unfortunately, DVG-profiler requires use of the HIVE software as described in its publication, which at the time of writing appears to be retired. We attempted to retrieve and compile the source code (https://github.com/kkaragiannis/DVG-profiler/), but due to version incompatibility with the Penn State ROAR Collab supercomputer, it could not be run and so was dropped from the study.

DG-seq is an R script published in 2020. It imports some functions and then processes user-assigned directories of .bam files. The imported functions consist of a function to import the .bam files and parse them into a data frame for downstream computing, one to create an uncertainty matrix for the reference (which is essentially a map of all short repeat sequences), one to run through the data frame and use CIGAR strings to identify possible junction points and then a normalization and filtering function that will only keep gaps greater than or equal to a user-defined value and are supported by a user-defined number of reads [34].

DVGfinder was published 2 years later (2022), combining the newest version of ViReMa available at the time (version 0.23) with DI-tector. It runs both programs on the input dataset, consolidating the results into a shared output with normalized terminology, noting which reads both programs reached consensus on and then applying a machine learning algorithm (trained on *in silico* generated datasets seeded with DVGs) to apply a ‘probability of being real’ value. It does not invent a new search program itself, merely combines two currently available and adds a new function [1].

Finally, VODKA2 (Viral Open-source DVG Key Algorithm 2) was published in 2023 and replaces the now discontinued VODKA [37], which could only detect copy-back DVGs, only screened the last 3,000 bp of the reference genome, did not validate via blast nor cluster DVG species and could not run multiple datasets in parallel. It is a set of shell scripts meant to be run in a Docker environment in a specific order. The first step is to create a ‘VODKA2 non-standard viral genome catalogue’, which is created from the reference genome and is a list of all possible combinations of break and rejoin along with *N* nt upstream of a break and downstream of a rejoin (this process can take up to 48 h or more depending on the size of the genome). These are written to a large multi-FASTA file as they are generated, and a bowtie2 index is then built on this multi-FASTA file and stored for later use. It accepts fastq input (it is recommended to trim and gzip them beforehand to help reduce data volume (E. Achouri, pers. commun.), and then, the VODKA2 analysis pipeline can begin, the first step being to use bowtie2 to align the reads against the genome. Then, reads are mapped to the catalogue, junction points are filtered through blast to remove all sequences with ambiguous blast alignment to the reference genome, junction points are aggregated into a ‘species’ designation and output tables are generated [36].

### Purpose of our meta-analysis

All these programs were created to identify DVGs by applying different methods to identify reads that do not align contiguously to the viral reference genome chosen. They report these as ‘junction points’, which correspond to the nt position on the full-length genome where the deletion starts (breakpoint) and ends (re-initiation point). As stated previously, efficient DVG discovery is important for robust viral quasispecies characterization as well as for the discovery of candidates for DVG-based antiviral therapies. High-throughput sequencing datasets have been suggested as beneficial for this rapid DVG discovery by providing a large amount of sequence data that can be screened bioinformatically, increasing efficiency. Several programs are available to aid with this: we wanted to provide a comparison between all currently available programs regarding the consistency of their outputs when run on the same datasets. A large driver for this interest is to identify defective candidates that can be screened downstream for utility as an antiviral: to be effective at this, the programs should have a low false-positive rate. Each program’s false-positive and false-negative rate has been reported in their respective publications; however, the test datasets are always *in silico* or experimental datasets produced by that lab. We wanted to assess whether, when used on a variety of other publicly available datasets across a variety of available virus/host combinations and methods of RNA-Seq, outputs from these different bioinformatic tools generally agree: to test this, we ran six datasets through five bioinformatic programs (ViReMa, DI-tector, DVGfinder, DG-Seq and VODKA2), with two controls: an experimental dataset produced by our lab and an *in silico* dataset published by Olmo-Uceda *et al*. [1]. We also ran a ‘program control’ where we just used bwa on the Penn State ROAR Collab supercomputer command line.

Furthermore, as stated previously, how common DVGs are in natural plant viral infections is still an open question, and high-throughput sequencing datasets, specifically ‘virome’ datasets and the bioinformatic tools used to process them, have been proposed as a resource to identify and characterize (downstream) the defective portion of the quasispecies. Our working hypothesis regarding DVG prevalence in virus populations is that if virally encoded proteins can assist genome replication in *trans* and that these *trans-*assisting virally encoded proteins increase the fitness of the genome they help replicate, DVGs should always be present in the viral quasispecies in the same host under the same conditions. We tested this by having three of our six tested datasets be ones where the host/virus combination has sequenced and literature-backed ‘canonical’ (experimentally proven) DVGs, and three where the virus is known to produce DVGs in other hosts but has not been assessed for DVGs in the sequenced host.

To explore these two questions – whether (and which) bioinformatic tools show promise for downstream DVG characterization and whether our working hypothesis on DVG prevalence has any virome data support – we performed a meta-analysis utilizing eight previously published datasets: three of a host/virus combination known to produce DVGs, three of a host/virus combination where the virus is known to produce DVGs in other hosts but the DVG status of the sequenced host is unknown and two controls, an RNA-Seq dataset of Severe Acute Respiratory Syndrome Coronavirus 2 (SARS-CoV-2)-infected Vero E6 cells treated with DVGs with known deletions and one *in silico* generated RNA-Seq dataset seeded with random DVG reads. Our results demonstrate a low degree of agreement regarding identified junction points between programs, including little agreement on the most commonly occurring junction point. However, the junction point(s) identified as ‘most common’ were typically large, disruptive deletions, meaning that they could be explored downstream for antiviral capability. Furthermore, we did find some support for our DVG prevalence hypothesis, with two out of three ‘known combination’ datasets finding canonical junction sites reported from other papers; however, all three of our ‘unknown combination’ datasets had canonical junction sites located as well.

## Methods

### Selection of datasets

The NCBI SRA database was searched for datasets of plants infected by each virus known to generate DVGs at the time of publishing, resulting in 12 possible viruses to choose from: *Tombusvirus cymbidii* [cymbidium ringspot virus (CymRSV)], *Betacarmovirus brassicae* [turnip crinkle virus (TCV)], *Pomovirus solani* (potato mop-top virus), *Nepovirus nigranuli* (tomato black ring virus), *Potexvirus flavitrifolii* [clover yellow mosaic virus (CYMV)], *Curtovirus betae* (beet curly-top virus), *Begomovirus manihotis* (African cassava mosaic virus), *Begomovirus manihotisafricaense* (East African cassava mosaic virus), *Cucumovirus CMV* [cucumber mosaic virus (CMV)], *Orthotospovirus tomatomaculae* [tomato spotted wilt virus (TSWV), *Tobravirus tabaci* (tobacco rattle virus) and *Bromovirus BMV* (brome mosaic virus, BMV). Of these, three datasets with literature-backed host–virus combinations were picked: CMV in *Nicotiana tabacum*, BMV in *Hordeum vulgare* and CymRSV in *Nicotiana benthamiana* (Table 1). Then, three test datasets containing viruses that are known to produce DVGs on other hosts, but which the sequenced host has not been tested, were selected. These include TCV in *Cicer arietinum*, CYMV in *Verbena officinalis* and TSWV in *Solanum lycopersicum* (Table 1). For positive controls, a synthetic dataset seeded with DVGs of known sequence was used [1], as well as two datasets consisting of SARS-CoV-2-infected Vero E6 cells treated with DVGs of known sequence produced by *in vitro* transcription.

**Table 1.** Information regarding datasets used in this meta-analysis In column 7, ‘breakpoint’ refers to the nt on the viral reference genome where the internal deletion begins, and ‘re-initiation point’ refers to the nt on the viral reference genome where the internal deletion ends. n/a means that there is no paper in the literature that supports either DVGs in this virus/host combination or that the virus generates DVGs, respectively.

Virus	Host	SRA run accession no. [publication]	Viral reference (NCBI)	RNA-Seq type, sequence platform	Paper(s) supporting DVGs in this virus+host	Paper(s) supporting that this virus generates DVGs	Junction used in analysis (breakpoint/re-initiation point)
*Bromovirus BMV* (BMV) RNA3	*Hordeum vulgare*	SRR10119525 [44]	NC_002028.2	Viral RNA-enriched RNA-Seq, Illumina HiSeq 2000	[45,46]	[45,46]	369/868 201/266 366/865
*Cucumovirus CMV* (CMV) RNA3	*Nicotiana tabacum*	SRR6374495_1 SRR6374495_2 SRR6374496_1 SRR6374496_2 [35]	AB008777	Transcriptomic RNA-Seq of host, Illumina HiSeq 2000	[47]	[47]	311/621 330/487
*Tombusvirus cymbidii* (CymRSV)	*Nicotiana benthamiana*	SRR039711 [48]	NC_003532	Viral small interfering RNA-Seq, Illumina Genome Analyzer	[3]	[3]	164/1351 1463/4327 164/1347 1504/4347 4407/4612
*Potexvirus flavitrifolii* (CYMV)	*Verbena officinalis*	SRR11928841_1 SRR11928841_2 SRR10490212_1 SRR10490212_2 [No pub.]	MT176428	Viral RNA-enriched RNA-Seq, Illumina NextSeq 500	n/a	[30]	757/6603 757/6681 814/6788 831/820
*Betacarmovirus brassicae* (TCV)	*Cicer arietinum*	SRR5571020 SRR5568966 [49]	NC_003821.3	Viral small interfering RNA-Seq, Illumina HiSeq 2500	n/a	[29]	141/3863
*Orthotospovirus tomatomaculae* (TSWV) segment L	*Solanum lycopersicum*	SRR12953073_1 SRR12953073_2 [50]	MN833246.1	Transcriptomic RNA-Seq of host, Illumina HiSeq 4000	n/a	[26,28,42, 43]	1031/6506 74/5681 138/5930
SARS-CoV-2	*Chlorocebus aethiops*	SRR35289193 SRR35289192 (No pub.)	NC_045512	PacBio long-read sequencing	DIs are synthetic	n/a	781/19664 20340/29902
*Potyvirus rapae* (TuMV)	*Brassica rapa* subsp. *rapa*	Dataset (*in silico* generated) in supplementary materials of [1]	GitHub of [1]	*In silico* generated dataset	DVGs are synthetic	n/a	24 deletion DI junctions, provided in the supplementary materials of [1]

The TSWV and CYMV strains that infected their respective datasets do not have a full sequence available on NCBI. Therefore, we tried to *de novo* assemble a TSWV L segment and CYMV genome from the dataset reads using Velvet 1.2.10 and VelvetOptimiser 2.2.6. We were unable to get full genome coverage and so could not use the *de novo* assembled genomes as a reference. However, we utilized blast with our partial genome contigs as query sequences to find the reference genome on the NCBI with the highest percent similarity. The chosen CYMV reference genome had 99.9% similarity to our *de novo* assembled partial genome, and the chosen TSWV segment L reference genome had 99.80% similarity.

### Workflow and software

All computing work was done on the ROAR Collab supercomputer at Penn State University on the ‘open’ account with standard cores, 1 node (4 cores per node), 1 core per task and 200 Gb memory in May–June 2024. Bioinformatic software was obtained from the following sources: https://www.sourceforge.net/projects/virema (ViReMa v0.29), https://www.di-tector.cyame.eu (DI-tector v0.6), https://rnajournal.cshlp.org/content/26/12/1905/suppl/DC1 [DG-seq, run on RStudio 2024.12.1+563 ‘Kousa Dogwood’ running R v4.3.2 (2023-10-31 ucrt) -- ‘Eye Holes’], https://github.com/MJmaolu/DVGfinder (DVGfinder v2) and https://github.com/lopezlab-washu/VODKA2 [VODKA2 (2.0)]. As previously mentioned, DVG-profiler [35] requires use of the HIVE software as described in its publication, which at the time of writing appears to be retired. The source code (https://github.com/kkaragiannis/DVG-profiler/) could not be run on Roar Collab due to unfixable version incompatibility, so DVG-profiler was dropped from the study.

Across all datasets with a read length of ~150 bp, ViReMa was run with the default seed of 25 nt (--Seed 25). Datasets with a read length of ~35 bp were run with a seed of 5 nt to be proportional to the longer read datasets. Other ViReMa settings were the use of 4 processors for parallel processing (--p 4) to enhance the speed of analysis and only counting deletions with a deletion length of 5 or greater (--MicroInDel_Length 5), which is the most often used cutoff when looking for defective genomes or recombination sites in plant viruses. All other settings were default. DI-tector was set to show only junctions with count reads greater than 5 (-n 5) for the same reasons as ViReMa, to skip alignments with indels smaller than or equal to 5 (-l 5) and to run on four threads for parallel processing (-x 4), with all other settings default. DVGfinder was also set to run on four threads (-n 4), with all other settings default (because you cannot alter ViReMa seed lengths when running DVGfinder, the ViReMa.py and ViReMa_config.py modules were edited to have a seed length of 5 for the datasets with read lengths of 35nt). VODKA2 was run as the scripts on GitHub (https://github.com/skybird99-anthony-taylor/DIs_in_RNAseq_data) indicate. DG-Seq required some tweaks to run on R v4.3.2, and the edited file with all its annotations is available on GitHub with the other scripts. All programs were run in a Miniconda3 environment ‘bioinformatics_env’ which had all programs and program dependencies (as indicated by program ReadMe files or error messages) downloaded into it via conda install.

As a control, bwa v0.7.18-r1243-dirty was installed to the ‘bioinformatics_env’ environment using pip. Bwa was selected due to the use of a dataset with long reads (the SARS-CoV-2 dataset), since the upper limit for read length for bowtie is ~1,000 bp, as described in the bowtie user manual. Bowtie2 does not have an upper limit, but the only program to use bowtie2 was VODKA2, while both ViReMa and DI-tector use bwa, so bwa was selected as the more relevant program to use. Datasets were downloaded from the SRA database using NCBI’s sra-toolkit (v3.1.0, centos linux 64 bit) into a ‘datasets’ sub-directory, and viral reference genomes corresponding to the SRA data’s publications (see Table 1) were also downloaded.

All SRA datasets were run through the fastqc command on default settings to check for quality and were filtered to remove all reads with quality scores <30 excepting reads from the SARS-CoV-2 datasets, which were generally between 2 and 30 and so had reads with quality scores <20 filtered out instead. If datasets were not pre-trimmed, adaptor sequences and primers were trimmed using cutadapt (default settings) based on the ‘overrepresented sequences’ and ‘adaptor content’ tabs in the fastqc report. Only overrepresented sequences that were flagged as an adapter were removed. SRA datasets were then aligned to the RefSeq genome and transcriptome of the plant host using bwa to filter out host reads and shrink datasets for downstream processing. All reads that did not align to the host genome or transcriptome were converted to a fastq.gz file for faster downstream processing. Control datasets consisted of *in vitro* transcribed RNA of a SARS-CoV-2 DVG transfected into Vero E6 cells (one dataset from cells before passaging and the other after three passages) as well as an *in silico* generated dataset with 72 random DVGs (24 deletion DVGs) of *Potyvirus rapae* (turnip mosaic virus, TuMV), each present in a proportion of 0.003 in regard to the total number of reads (which included both TuMV and reads from the ‘host’, *Brassica rapa* subsp. *rapa*).

### Analysis

For ‘known’ virus/host combinations, junctions used for analysis were taken from the supporting paper (Table 1). For ‘unknown’ virus/host combinations, junctions used for analysis were taken from papers with sequences reported for that virus’ DVGs. This was reference [29] for TCV, reference [30] for CYMV, and reference [27] for TSWV. Considering that the junctions that make up a particular virus’ DIs are often heterogeneous, a breakpoint (BP, referring to the nt on the viral reference genome where the internal deletion begins) and/or re-initiation point (RI, referring to the nt on the viral reference genome where the internal deletion ends) shift of up to 5 nt in either direction (10 nt total) was tolerated to count a junction as a positive result. R scripts were written to search through the large output files for junctions, with the 10 nt interval. Considering that in plants, deletion DVGs are the most well-studied, and all the canonical DVGs chosen to search for in the chosen datasets are deletion DVGs, in programs that gave multiple DVG outputs (e.g. insertion, deletion and copy-back), only deletions were analysed. R code for analysis can be found at the project’s GitHub. DG-Seq is an R program already, so R code was not written for this analysis: however, as previously stated, it required some debugging to run, and the fixed code can be found on the GitHub. VODKA2 was originally created to run in a Docker environment and does not function properly without Docker (which cannot be installed into Roar Collab), so also provided in the GitHub is an example .txt file of code with instructions detailing how to edit and run the VODKA2 scripts to get it to function in a non-Docker environment. These fixes were a mix of those discovered during analysis by A. Taylor and those suggested by VODKA2’s author, E. Achouri.

For the manual alignment using bwa, the reference file was the junction point itself instead of the whole virus genome. This was done by taking the junction point coordinate of the canonical DVG reported in their respective publications (columns 5 and/or 6 from Table 1), locating this coordinate on the viral reference genome that we were using for that dataset, and selecting 20 nt on either side of that coordinate to create a ‘junction point reference’ sequence, which was then used as the reference to align samples against. We checked mapped (-f 4), secondary mapped (-f 256) and split mapped (-f 2048) reads by hand against both the DVG junction reference genome and full virus reference genome using SnapGene v7.2.1, with only 99–100% matches considered a hit. Junction reference files can be found at the project’s GitHub. The results for this portion of the analysis are given in Table 2.

**Table 2.** Table summarizing the results for canonical DVG junction search across datasets and programs Column 1 gives the virus/host analysed, with the total number of reads analysed for that virus/host combination. Columns 2–7 give program-specific data. In each column/row intersection, there are four lines of information. The first line reports how many canonical junctions out of the canonical junctions listed in Table 1 were found (e.g. 1/3). *The second line reports the BP/RI of these junction(s) (e.g. 201/266). The third line gives the total number of output rows provided in the programs’ results table (e.g. 1,017 total). This number is not adjusted in any way, meaning that the same junction may be counted multiple times. The final line reports the adjusted number of junctions found, where junctions are appropriately binned and duplicates are removed, resulting in a final count of ‘unique’ junctions with no redundancy.*

Virus/host (total reads analysed)	No. of known junctions found (BP/RI (genome position), if found) Total no. of output lines given by a program [Total no. of unique junction observations (removal of redundancies)]					
	***DI-tector***	***ViReMa***	***DVGfinder***	***DG-seq***	***VODKA2***	***BWA***
BMV/*H. vulgare* (54,465,018 reads)	1/3 (201/266) 1,017 total [120 unique]	1/3 (201/266) 295,555 total [40,905 unique]	1/3 (201/266) 2,832 total [587 unique]	1/3 (201/266) 1,520 total [1520 *unique]*	0/3 326 total [96 unique]	1/3 (201/266) 527 total
*CMV/N. tabacum* *(77,842,721 reads)*	0/2 0 total	0/2 1,979 total [1810 unique]	0/2 0 total	0/2 21 total [13 unique]	0/2 45,541 total [10,756 unique]	0/2 0 total
*CymRSV/N. benthamiana* *(1,814,303 reads)*	0/5 168 total [94 unique]	4/5 (1,463/4,327, 1,64/1,347, 1,504/4,347, 4,407/4,612) 166,036 total [162,395 unique]	0/5 94 total [94 unique]	0/5 0 total	0/5 3 total [3 unique]	0/5 0 total
*CYMV/V. officinalis* *(102,793,765 reads)*	0/4 40 total [31 unique]	1/4 (831/820) 91,035 total [80,216 unique]	2/4 (757/6,608, 831/820) 501,250 total [445,032 unique]	0/4 427 total [366 unique]	0/4 221 total [187 unique]	0/4 0 total
*TCV/C. arietinum* *(11,261,556 reads)*	0/1 0 total	1/1 (141/3,863) 152,027 total [92,672 unique]	0/1 6,274 total [4,024 unique]	0/1 0 total	0/1 1,045 total [657 unique]	0/1 0 total
*TSWV/S. lycopersicum* *(27,028,502 reads)*	0/3 0 total	0/3 9 total [8 unique]	2/3 (74/5,681, 138/5,930) 226,516 total [226,016 unique]	0/3 2 total [2 unique]	0/3 5 total [5 unique]	0/3 0 total
*SARS-CoV-2/C. aethiops* *(100,898 reads)*	0/2 220 total [84 unique]	0/2 306 total [264 unique]	0/2 0 total	0/2 0 total	0/2 0 total	2/2 (781/19,664 20,340/29,902) 3,509 total
*TuMV/B. rapa subsp. rapa* *(100,001 reads)*	11/24 53 total [24 unique]	16/24 95 total [95 unique]	29/24 29 total [29 unique]	34/24 35 total [34 unique]	8/24 44 total [27 unique]	24/24 100,099 total

**Fig. 1.**
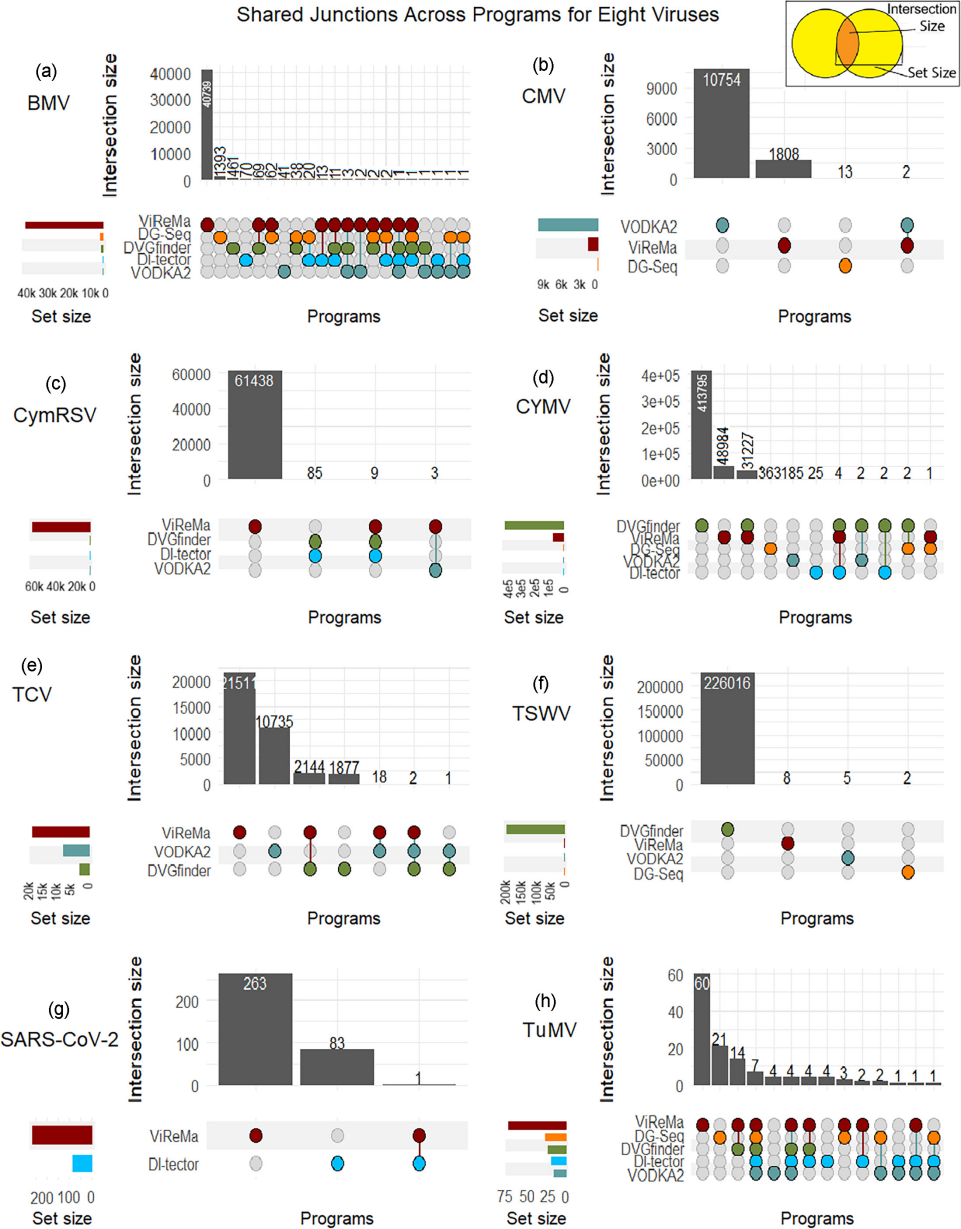
UpSet plots showing the degree of overlap between programs regarding junctions identified and written to their outputs. Panels (a, b, c, d, e, and f) correspond to the test datasets BMV, CMV, CymRSV, CYMV, TCV, and TSWV, respectively. Panels (g) and (h) correspond to the two control datasets, SARS-CoV-2 and TuMV. BMV (**a**) showed the most complex degree of overlaps, while TSWV (**f**) showed no overlaps. Control datasets are SARS-CoV-2 (**g**) and TuMV (**h**).

For the determination of junction overlap across programs (Fig. 1), compiled datasets were created by taking all the breakpoint (BP) and re-initiation point (RI) values provided in the programs’ outputs and compiling them in a new .csv file with a third column of ‘program’ indicating which program’s output that junction was from. A fourth column of ‘ID’ was created whereby the BP and RI from each junction was combined into one character separated by an underscore (e.g. for a junction of 300/3,381, the BP of 300 and RI of 3,381 would become ID 300_3381). These combined datasets were then imported to R 4.3.2 and visualized as UpSet plots using the package ComplexUpset 1.3.6.

For the investigation into the most frequently reported junction in the program outputs (Table 3), R codes were written to count all instances of a particular junction in the output and then list them in descending order. This list was then manually evaluated and, if there was a cluster of closely related junction points (e.g. 61, 54 and 46 respective instances of junctions 152/52, 152/40 and 152/48), they were grouped together for final instance counts and given an ‘average’ junction value (e.g. 152/47 for the previous example). A breakdown of which junction points this was done for, along with what junction points were included in the grouping, is given in Table S2 (available in the online Supplementary Material). This instance count was then divided by the total number of DVGs found by the program for that dataset to provide a percent-of-output value. Note that for this analysis, instances where no junctions were found more than one time were not counted, as were lists of junctions greater than ten with tied hits, although the latter is provided in Table S3. The deletions caused by the most commonly reported junction points were then re-created on SnapGene v7.2.1 by opening the viral genome reference sequence, hand-annotating this reference based off the most recent NCBI annotations for that virus, and then deleting the nt between the BP and RI (e.g. for a junction of 300/3,381 and nt 301–3,380 would be deleted). The effects on the annotated features, specifically ORF length (in nt), were noted in Table 3. We considered a ‘substantial effect’ to be a deletion of at least one ORF, a frameshift of at least one ORF or a truncation of an ORF greater than 60 nt.

**Table 3.** Report on the most common junction site reported by each bioinformatic program, along with information on percent total instances in the output files and information regarding what influence that junction has on genomic organization Instances where no junctions were found more than one time were not counted, as were lists of junctions greater than ten with tied hits. Consult Table S2 for a list of what junctions went into a bin and Table S3 for the full list of junctions greater than ten with tied hits. MP, movement protein; CP, coat protein; RdRp, RNA-dependent RNA polymerase; SS, silencing suppressor; TGB, triple gene block protein.

Program	Virus/*host*	Most commonly reported binned junction(s)	No. of observations in that binned junction (% of total observations)	Affected genomic portion (protein encoded)	Disruption description	Found in other programs?
DI-tector	CymRSV/*N. benthamiana*	691/3024	47 (28.0)	ORFs 1 and 2 (RdRp, CP)	Creates a new 684 nt ORF1 and truncates ORF2 by 553 nt	Yes [DVGfinder (only DI-tector)]
DI-tector	SARS-CoV-2/*C. aethiops*	60/28250	122 (55.5)	All ORFs except ORF9 (CP)	Removes all ORFs except for ORF9 (CP), which is unaffected	Yes (ViReMa)
DI-tector	TuMV/*B. rapa*	2964/6926	7 (29.2)	ORF1 (polyprotein)	Removes the coding sequence for the polypeptides 6K1, CI, 6K2 and NIa-VPg and truncates polypeptides P3 and NIa-Pro	Yes [ViReMa, DVGfinder (both DI-tector and ViReMa), VODKA2]
DI-tector	CYMV/*V. officinalis*	3927/6856	5 (12.5)	ORFs 1, 2, 3 and 4 (replicase, MP, TGB, CP)	Removes ORFs 2, 3 and 4; truncates ORF1 by 1,104 nt	No
ViReMa	BMV/*H. vulgare*	240/300	43 (0.015)	ORF1 (MP)	Truncates ORF1 by 63 nt	No
ViReMa	CMV/*N. tabacum*	38/16	10 (0.62)	5′ UTR	No effect on ORFs, effects RNA secondary structure (based on RNAfold webserver)	No
ViReMa	CymRSV/*N. benthamiana*	3349/3213 3465/4136 4604/4136 4373/4136 698/2590 3500/4076	3 (0.002) 3 (0.002) 3 (0.002) 3 (0.002) 3 (0.002) 3 (0.002)	ORF2 (CP) ORFs 2, 3 and 4 (CP, MP, SS) ORFs 3 and 4 (MP, SS) ORFs 3 and 4 (MP, SS) ORF1 (RdRp) ORFs 2, 3 and 4 (CP, MP, SS)	Truncates ORF2 by 137 nt; removes ORFs 3 and 4 completely; truncates ORF 2 by 297 nt; truncates ORF3 by 231 nt and ORF4 by 174 nt; truncates ORF3 by 171 nt and ORF4 by 219 nt; truncates ORF1 by 1,893 nt; creates a new 1,158 nt ORF2 and a 249 nt ORF3	No No No No No No
ViReMa	SARS-CoV-2/*C. aethiops*	29871/29890	22 (7.19)	3′ UTR	Poly-A tail truncation	No
		2640/910	4 (0.004)	ORF1 (replicase)	Truncates ORF1 by 1,731 nt	No
		1123/1117	4 (0.004)	ORF1 (replicase)	Splits ORF1 into two smaller ORFs, with the 5′ ORF (1,026 nt) in the same frame (+2) and the 3′ ORF (3,924 nt) frameshifted to +1	No
		1213/2761	4 (0.004)	ORF1 (replicase)	Splits ORF1 into two smaller ORFs, with the 5′ ORF (1,200 nt) in the same frame (+2) and the 3′ ORF (2,388 nt) frameshifted to +1	No
ViReMa	CYMV/*V. officinalis*	559/4093	4 (0.004)	ORF1 (replicase)	Splits ORF1 into two smaller ORFs, with the 5′ ORF (540 nt) in the same frame (+2) and the 3′ ORF (939 nt) frameshifted to +1	No
		1717/1819	4 (0.004)	ORF1 (replicase)	Splits ORF1 into two smaller ORFs, with the 5′ ORF (1,728 nt) in the same frame (+2) and the 3′ ORF (2,859 nt) frameshifted to +1	No
		1499/6849	4 (0.004)	All ORFs	Truncates ORF1 by 3,615 nt, removes all other ORFs	No
		1706/239	4 (0.004)	ORF1 (replicase)	Splits ORF1 into two smaller ORFs, with the 5′ ORF (363 nt) in the same frame (+2) and the 3′ ORF (3,384 nt) frameshifted to +1	No
		6493/6538	4 (0.004)	ORF4 (CP)	Truncates ORF4 by 237 nt	No
ViReMa	TSWV/*S. lycopersicum*	1124/1042	2 (22.22)	ORF1 (RdRp)	Truncates ORF1 by 1,110 nt	No
DVGfinder	BMV/*H. vulgare*	765/762	66 (2.33)	ORF1 (MP)	Truncates ORF1 by 120 nt	Yes (DI-tector, ViReMa, VODKA2)
DVGfinder	CymRSV /*N. benthamiana*	695/3024	8 (8.51)	ORFs 1 and 2 (RdRp, CP)	Creates a new 1,281 nt ORF1	Yes (DI-tector, ViReMa)
DVGfinder	TCV/*C. arietinum*	1452/249 1449/2431 1452/3151 1452/3891	4 (0.064) 4 (0.064) 4 (0.064) 4 (0.064)	ORFs 1 and 2 (Aux. RdRp, RdRp) ORF 2 (RdRp) ORFs 2, 3 and 4 (RdRp, MP, CP) ORFs 2, 3 and 4 (RdRp, MP, CP)	Truncates ORF1 by 528 nt and ORF2 by 624 nt and moves ORF2 from frame +1 to +3; truncates ORF2 by 798 nt; truncates ORF2 by 924 nt, removes ORF three completely and truncates ORF4 by 420 nt; truncates ORF2 by 420 nt, removes ORFs 3 and 4 completely	Yes (ViReMa) Yes (ViReMa) Yes (ViReMa) Yes (ViReMa)
DVGfinder	CYMV/*V. officinalis*	719/5103	5 (0.0001)	ORFs 1 and 2 (RdRp, MP)	Truncates ORF1 by 4,380 nt and ORF2 by 24 nt	No
		3854/627	3 (0.001)	ORF1 (RdRp)	Truncates ORF1 by 3,248 nt	No
DVGfinder	TSWV/*S. lycopersicum*	3754/4156	3 (0.001)	ORF1 (RdRp)	Splits ORF1 into two ORFs of 3,744 and 4,449 nt	No
		719/5103	3 (0.001)	ORF1 (RdRp)	Splits ORF1 into two ORFs of 687 and 3,609 nt	No
VODKA2	BMV/*H. vulgare*	1239/1245	72 (22.1)	Intergenic region	No effect	No
VODKA2	CMV/*N. tabacum*	670/1971	800 (1.76)	ORFs 1 and 2 (3a, CP)	Truncates ORF1 by 84 nt, removes ORF2 completely	No
VODKA2	TCV/*C. arietinum*	193/3688	8 (0.766)	All ORFs	Removes all ORFs	No
VODKA2	CYMV/*V. officinalis*	234/5505	9 (4.07)	ORFs 1 and 2 (replicase, MP)	Creates a new 432 nt ORF1, removes ORF2	No
VODKA2	TuMV/*B. rapa*	2964/6926	6 (13.6)	ORF1 (polyprotein)	Removes the coding sequence for the polypeptides 6K1, CI, 6K2 and NIa-VPg and truncates sequence for polypeptides P3 and NIa-Pro	Yes [DI-tector, ViReMa, DVGfinder (both DI-tector and ViReMa)]
DG-seq	BMV/*H. vulgare*	198/265	432 (28.42)	ORF1 (MP)	Truncates ORF1 by 273 nt	Yes (ViReMa)
DG-seq	CMV/*N. tabacum*	870/874	7 (33.33)	ORF1 (3 a)	Extends ORF1 by 93 nt	No
DG-seq	TuMV/*B. rapa*	6000/6040	2 (5.71)	ORF1 (polyprotein)	Splits the polyprotein ORF into two ORFs in the middle of the section encoding the NIa-Vpg protein; deletes 14 aa	Yes (DI-tector, DVGfinder, ViReMa, VODKA2)

## Results

### Canonical DVG junction sites were not consistently found by bioinformatic programs

The first question we explored was whether canonical junction sites reported in previous papers could be found in other RNA-Seq datasets of the same host/virus combination. Table 2 summarizes this search in the eight analysed datasets. Column 1 gives the virus/host analysed, with the total number of reads analysed for that virus/host combination. Columns 2–7 give program-specific data. In each column/row intersection, there are four lines of information. The first line reports how many canonical junctions out of the canonical junctions listed in Table 1 were found (e.g. 1/3). The second line reports the BP/RI of the first line’s found junction(s) (e.g. 201/266). The third line gives the total number of output rows provided in the programs’ results table (e.g. 1017 total). This number is not adjusted in any way, meaning that the same junction may be counted multiple times. The final line reports the adjusted number of junctions found, where junctions are appropriately binned and duplicates are removed, resulting in a final count of ‘unique’ junctions with no redundancy. As an example, if a TCV dataset run through DI-tector gives 10 lines of output, with those output junctions being 141/3862, 142/3863, 280/300, 100/750, 101/754, 2325/3543, 1124/2534, 3701/3446, 2022/2100 and 2236/1756, then in Table 2 under the TCV/DI-tector row/column intersection, the first line would read 1/1 (since the junction 141/3862, the canonical junction site referenced in Table 1, was found), the second line would read 141/3862 (to clarify which canonical junction was found, since many combinations have more than one junction searched for), the third line would read *10 total* (for 10 total lines) and the last line would read *[8 unique]* because 141/3862 and 142/3863 are two instances of the same junction within the 10 nt wiggle room, as is 100/750 and 101/754. The differences in script (e.g. underline, italics and brackets) are to help better differentiate the different lines of information and have no encoded meaning.

Of the three virus/host combinations that have been previously shown to produce DVGs (‘known’ combinations), only two had previously published DVG junctions identified in the RNA-Seq datasets used: BMV in *H. vulgare* and CymRSV in *N. benthamiana*. BMV had one junction (201/266) identified by DI-tector, ViReMa, DVGfinder, DG-seq and bwa (our ‘control search’), while CymRSV junctions were only found in ViReMa. A second junction of BWV, 875/362, is very close to the canonically reported one and was reported by several programs but was outside of the 10 nt wiggle room and so was not counted as per our methods. Interestingly, all three datasets involving a known DVG-producing virus in a novel host (‘unknown’ combinations) had a junction corresponding to canonical DVGs from that virus, although there was no consensus between programs. In TCV, ViReMa identified deletions corresponding to the known junction site 141/3863. In CYMV, ViReMa identified deletions corresponding to the known junction site 831/820, and DVGfinder found 831/820 as well as 757/6608. In TSWV, DVGfinder found deletions corresponding to the known junction sites 74/5681 and 138/5930. It is worth noting that the only canonical junction site found by our ‘control search’ (bwa) against the sequence of the junction was the 201/266 junction from BMW as well as the junctions in our control datasets.

Considering the control datasets, the TuMV dataset that was *in silico* generated saw all programs find junctions. However, all programs except bwa found far fewer than the 24 deletion junctions purposely seeded into the dataset. VODKA2 found the fewest seeded deletions, at only eight. DVGfinder and DG-seq found more junction sites than were seeded, meaning that five and ten of those junction sites were false positives, respectively. TuMV results are not listed in Table 2 due to length but can be found in Table S1. Bwa found all 24 with no false positives. The SARS-CoV-2 dataset had no programs except bwa find the two junctions present in the synthetic DVGs.

In sum, Table 2 demonstrates that two out of three known DVG-producing host/virus combination datasets saw at least one bioinformatic program find canonical DVGs, and all three unknown DVG-producing host/virus combination datasets had canonical DVG junction points found.

### There is little overlap in results between programs

Fig. 1 demonstrates the overlap in output between all the programs for each dataset. ViReMa and VODKA2 generally had the highest amount of output, numbering in the tens to hundreds of thousands of unique junction points, although DVGfinder had the highest output (from the ViReMa portion of the model) in CYMV and TSWV. Despite this, there is always very little overlap between the programs. There are two other main points of interest: one, that in four of the six datasets, DI-tector identified no deletions and so is not present (Fig. 1b, e and f); and two, that in four cases – BMV, CYMV, TCV and TSWV (Fig. 1a, d, e and f) – DVGfinder identified junctions that were not found in either DI-tector or ViReMa, which is unusual considering that DVGfinder’s search algorithm is made up of those two programs. In the case of TCV, DVGfinder ascribes these junctions to ViReMa, for it records no instances of a DI-tector identification for any of its output junctions. In the case of BMV and CYMV, DVGfinder ascribes some of these junctions to ViReMa, some to DI-tector and some to both. For TSWV, all the DVGfinder junctions are unique to DVGfinder. Therefore, our results show that in some cases, DVGfinder writes junctions to output that are not found by ViReMa or DI-tector when those programs are run independently. For ViReMa, this may be because of version differences: DVGfinder uses v0.23, while our independent run uses v0.29. Version difference cannot be the explanation for DI-tector, since the versions used between DVGfinder and our independent runs are the same. Fig. 1 also demonstrates that in most datasets, there were no junctions found by all programs, except TCV and the control TuMV. For TCV, all programs that gave results for this dataset find junction 692/1234, which fuses ORFs 1 and 2, and junction 1616/2191, which truncates ORF2. For TuMV, consensus junctions were 6000/6040, 3859/7242, 1784/6060, 3516/5147, 1909/8273 and 1051/2302 – all true positives. None of these junctions were the most common junction found in the datasets as described in Table 3, except for 6000/6040 of the TuMV dataset, which was the most common junction in the DG-Seq output. Considering that all the true positives seeded into the TuMV dataset were seeded into it in the same proportion (0.003), it is interesting that one was found more often than the other 23.

### There is little agreement regarding the most common junction point in a dataset across programs

The second major question we addressed was whether the outputs from the different bioinformatic tools agreed when the same dataset was run through them. Fig. 1 soundly demonstrates that for the most part, the outputs do not agree; however, we also wanted to know if those that do all identify the same junction as the most common. Table 3 demonstrates that the most commonly occurring junction site(s) identified by different programs also generally does not agree. The only junction site identified as the most common between two programs was junction 2964/6926 from TuMV, which was identified as the most common in that dataset’s DI-tector output as well as VODKA2.

Some junctions that were identified as the most common under one program were also in the outputs of others, just not as the most common. As Table 3 demonstrates, this is mostly due to DVGfinder overlapping with DI-tector and ViReMa, which is to be expected considering DVGfinder’s design. However, the BMV junction 765/662 (most common in DVGfinder from DI-tector) was found in ViReMa and VODKA2’s outputs, and the BMV junction 198/265 (most common in DG-Seq) was found in ViReMa’s output. This also occurred with the TuMV control junctions 2964/6926 (most common in DI-tector and VODKA2, also found in ViReMa and DVGfinder) and 6000/6040 (most common in DG-Seq, also found in DI-tector, DVGfinder, ViReMa and VODKA2) and the SARS-CoV-2 control junction 60/28250, which was the most common junction point in DI-tector but was also present in low levels in the ViReMa output. Interestingly, DVGfinder did not locate this junction point at all.

### Some identified DVGs would be worth exploring downstream for interfering capability, while others are most likely neutral mutations

One of the many reasons to use bioinformatic programs to look at the defective portions of the virome is the discovery and characterization of DI RNA (or DNA) that can be used as an antiviral against a virus of interest. Assessment for interfering capability of a DVG identified in a bioinformatic workflow must be done downstream, but in many circumstances, thousands of unique DVGs are identified in the output, so a method must be developed to identify a testable number of good candidates (Table 2). As previously stated, many known DI RNAs are present in high enough abundance that they can be seen as separate bands on a Northern blot [25,30]. Therefore, the most common junction point could correspond to a DI and be an effective starting point for downstream testing. We assessed whether the most common junction point showed promise for downstream validation by taking the most common junction points in Table 3, re-creating the deletions in the viral reference genome in SnapGene v7.2.1 (further described in Methods in the ‘Analysis’ section, final paragraph) and observing the effects of the deletion on genome organization. The most common deletions typically corresponded to ORF truncation or deletion, sometimes completely reshuffling genomic organization, like the junction point 193/3688 found by VODKA2 in TCV/*C. arietinum*. However, some common junctions, such as 29871/29890 (ViReMa, SARS-CoV-2/*C. aethiops*) and 1239/1245 (VODKA2, BMV/*H. vulgare*), have no effect on genome organization.

## Discussion

### Our results and DVG prevalence theory

Our working hypothesis regarding DVG prevalence in virus populations is that if virally encoded proteins can assist in genome replication in *trans* and that these *trans-*assisting virally encoded proteins increase the fitness of the genome they help replicate, DVGs should be always present in the viral quasispecies: something previously observed in viroids [38,40]. Hence, DVGs should be consistently found in all infections caused by that same virus species/host species combination under the same conditions. We tested this hypothesis by choosing three datasets that came from a virus/host combination that has been previously shown in the literature to generate DVGs, and in 2/3 cases, our hypothesis was not disproved. However, the third dataset, CMV/*N. tabacum*, did not support our prediction. This could be explained by the fact that the strain of CMV used in the experiment that generated the dataset was different than the strain known to produce DVGs.

Given our working hypothesis, we also predicted that we likely would not find previously published DVG junctions in novel hosts, since the fitness landscape of the viral population in that host would lead to DVGs of a different genome organization, if that host is permissive. However, all our ‘unknown’ combination datasets had canonical junction sites found. This is interesting because it suggests that perhaps for some viruses, the same DVGs can be generated irrespective of host species, something that previously did not seem to be true [28,41]. However, considering that previous research on TCV, TSWV and CYMV DVGs has found them in high enough abundance to get clear Northern blots [26,30,42, 43] but that the junctions we found bioinformatically areis nowhere near the most commonly occurring junction point; that ViReMa (the one responsible for identifying the junctions, even in the DVGfinder output) does produce false positives [32]; and that our control alignment of aligning the dataset to the sequence of the junction point did not identify the ‘found’ junctions, leads us to be sceptical of the validity of the identification. However, we also acknowledge that this could be biased in favour of our own hypothesis. Regardless, we conclude that we appear to have preliminary support for our hypothesis, but it is clear that more investigation needs to be done.

### Variability in program output

Running the same dataset through different programs and getting some differences in output has been previously noted between ViReMa and DG-seq [34]. Ergo, our results to this end are consistent with the literature, although we are the only paper to our knowledge to compare outputs from a single dataset across *all* currently available bioinformatic pipelines. The results of this, as Fig. 1 shows, are that there is little overlap between program outputs: even DVGfinder does not overlap completely with DI-tector and ViReMa outputs when those two are run independently. This indicates that the choice of program used for analysis matters, because it can influence the resulting conclusions drawn from a dataset. We suggest utilizing more than one program when analysing a dataset, to get multiple ‘points of view’ and a thorough analysis of the data.

It is worth considering that the method of sequencing, including RNA extraction, library prep and the sequencer used, likely influences program outputs as well. For example, for most of the datasets, ViReMa produces the largest output, but for CMV, it is VODKA2 and CYMV/TSWV and DVGfinder (primarily through ViReMa). However, we currently have no hypothesis as to what sequencing parameters may influence this. There is no clear pattern of association between sequencing platforms or library prep methods with size of output by different programs.

Since our search parameters for all the programs were relatively loose, many of the outputs were in the thousands or higher (Table 2). Since our Table 3 results show that for the most part the most frequently observed junctions correspond to substantial genome organization changes (and so are generally worth downstream screening for interfering capabilities), it probably is worth using stricter parameters for ViReMa and/or VODKA2. However, for some of the programs, even the loose parameters we used gave no results, meaning that stricter parameters would have been useless. We suggest that if multiple programs are utilized as previously suggested, an iterative approach to analysis may be most effective: starting with looser parameters and then getting stricter once preliminary results are back. It would be an interesting future investigation to see if increasing the number of replicates (e.g. running ten TSWV/*S. lycopersicum* RNA-Seq datasets prepared and sequenced in the same way) would allow for better estimation of background noise and more meaningful pattern searching.

### Analysis drawbacks

The analysis presented here does have several weak points that should be addressed. For one, although comparing datasets from multiple labs does adequately address our hypothesis of DVG prevalence, our conclusions would be more robust if all the datasets were the same type of RNA-Seq and were created (extraction, enriching, library prep and sequencing) the same way, to minimize the influence of sequencing. Therefore, it could be argued that they are not directly comparable. However, regardless of the high number of high-throughput datasets available, the number of datasets that can be used to investigate our DVG prevalence hypothesis is low because there are not many plant systems with verified DVGs available as high-throughput sequencing datasets. Furthermore, our hypothesis is not contingent on a direct comparison, and a single dataset can have its results from the various programs directly compared.

One major drawback of using next generation sequencing for this kind of DVG investigation that needs addressing is the inability to identify small mosaic genomes, which, at least in plants, tend to be the type of DVGs that alter infection dynamics enough to be noticed and characterized in all the previously cited literature. To identify these genomes, one needs long-read sequencing data, of which our SARS-CoV-2 dataset was, but none of the programs used are optimized to analyse long reads. Both DI-tector and ViReMa are capable of it, but neither program identified the junction points of the synthetic DVG present in that dataset, which was a small 2,883 bp mosaic genome of SARS-CoV-2 (NC_045512) (Table 2). Its sequence can be found in this paper’s GitHub.

Regarding controls, it is interesting to note that even the *in silico* generated dataset failed to have 100% consensus across all programs. In fact, for the TuMV dataset, both DG-seq and DVGfinder (the program that dataset was designed for) found false positives (Table 2). Only bwa found the exact number of seeded junction sites with no false positives or negatives. However, since the references we used for the bwa ‘control’ search were the sequences of the junctions themselves, we stress that although this method is a good validation to confirm the presence of a junction found by another program, it cannot be used to identify any novel DVGs.

## Conclusions

In this paper, we explored the utility of currently available bioinformatic programs for identifying junction points in HTS data that could be tested downstream for antiviral capacity. Specifically, we looked at whether the outputs from these bioinformatic tools generally agree. We also explored the possibility that these tools could help us address a larger research question of whether DVGs are consistently generated and maintained in a specific virus–host combination when conditions are permissive for their replication and accumulation, our ‘prevalence’ hypothesis. Our results demonstrate a low degree of agreement regarding identified junction points between programs, promise for finding the most commonly occurring DVG junctions and minor support for our prevalence hypothesis. We suggest the use of multiple programs on a dataset to better inform decisions regarding deletions to re-create and screen downstream and reiterate the importance of other avenues of evidence in DVG characterization.

## Supplementary material

10.1099/jgv.0.002176 Uncited Supplementary Material 1.

## Abbreviations

BMV: brome mosaic virus
bwa: Burrows–Wheeler alignment
CMV: cucumber mosaic virus
CymRSV: cymbidium ringspot virus
CYMV: clover yellow mosaic virus
DI: defective interfering
DVG: defective viral genome
HIVE: High-performance Integrated Virtual Environment
RT-qPCR: reverse transcription quantitative polymerase chain reaction
SARS-CoV-2: Severe Acute Respiratory Syndrome Coronavirus 2
TBSV: tomato bushy stunt virus
TCV: turnip crinkle virus
TSWV: tomato spotted wilt virus
TuMV: turnip mosaic virus
ViReMa: Viral Recombination Mapper
VODKA2: Viral Open-source DVG Key Algorithm 2
