# Supplementary material for: Defective but promising: evaluating the utility of currently available bioinformatic pipelines for detecting defective viral genomes in RNA-Seq data
Source: J Gen Virol. 2025 Nov 17;106(11):002176. doi: 10.1099/jgv.0.002176 (PMC12622790; doi:10.1099/jgv.0.002176)
Supplement: Uncited Supplementary Material 1. [file jgv-106-02176-s001.pdf]

SUPPLEMENTAL TABLE 1
A list of all canon junctions found by all programs for TuMV

Junction	Program
1051/2302	DG-SEQ
1784/6060	DG-SEQ
1909/8273	DG-SEQ
1973/1071	DG-SEQ
2962/6924	DG-SEQ
3000/8254	DG-SEQ
3516/5147	DG-SEQ
3792/3598	DG-SEQ
3859/7242	DG-SEQ
4022/7019	DG-SEQ
4060/2926	DG-SEQ
4165/6104	DG-SEQ
4275/1502	DG-SEQ
4479/8665	DG-SEQ
4531/4795	DG-SEQ
4559/4653	DG-SEQ
4844/3611	DG-SEQ
49/6092	DG-SEQ
490/176	DG-SEQ
5065/8595	DG-SEQ
5215/4600	DG-SEQ
5741/1803	DG-SEQ
5891/9640	DG-SEQ
6000/6040	DG-SEQ
6000/6040	DG-SEQ
704/6225	DG-SEQ
742/6504	DG-SEQ
7573/5083	DG-SEQ
7826/3885	DG-SEQ
7883/2983	DG-SEQ
8365/7470	DG-SEQ
8579/1434	DG-SEQ
8648/9347	DG-SEQ
8779/3087	DG-SEQ
8831/918	DG-SEQ
1784/6060	DI-TECTOR

2366/3648	DI-TECTOR
2964/6926	DI-TECTOR
4019/7017	DI-TECTOR
4022/7019	DI-TECTOR
4166/6105	DI-TECTOR
4533/4797	DI-TECTOR
5016/8357	DI-TECTOR
55/8684	DI-TECTOR
6000/6040	DI-TECTOR
745/6507	DI-TECTOR
8650/9349	DI-TECTOR
1004/7321	DVGFINDER
102/4015	DVGFINDER
1040/8978	DVGFINDER
1051/2302	DVGFINDER
1071/1973	DVGFINDER
1460/2998	DVGFINDER
1502/4275	DVGFINDER
163/4932	DVGFINDER
1707/7655	DVGFINDER
176/490	DVGFINDER
1784/6060	DVGFINDER
1803/5741	DVGFINDER
1909/8273	DVGFINDER
1973/1071	DVGFINDER
2354/4290	DVGFINDER
2366/3648	DVGFINDER
2387/1501	DVGFINDER
2677/3537	DVGFINDER
2964/6926	DVGFINDER
2983/7883	DVGFINDER
3000/1458	DVGFINDER
3002/8256	DVGFINDER
3200/9054	DVGFINDER
3324/6240	DVGFINDER
3409/4133	DVGFINDER
3497/970	DVGFINDER
3516/5147	DVGFINDER
3537/2677	DVGFINDER
3598/3792	DVGFINDER
3611/4844	DVGFINDER
3792/3598	DVGFINDER
3859/7242	DVGFINDER

3885/7826	DVGFINDER
4016/101	DVGFINDER
4022/7019	DVGFINDER
4062/2928	DVGFINDER
4133/3409	DVGFINDER
4255/7271	DVGFINDER
4275/1502	DVGFINDER
4560/4654	DVGFINDER
4600/5215	DVGFINDER
4653/4559	DVGFINDER
4795/4531	DVGFINDER
4846/3613	DVGFINDER
490/176	DVGFINDER
4964/8435	DVGFINDER
5083/7573	DVGFINDER
5147/3516	DVGFINDER
5149/8484	DVGFINDER
552/5442	DVGFINDER
5741/1803	DVGFINDER
5893/9642	DVGFINDER
6000/6040	DVGFINDER
6040/6000	DVGFINDER
6104/4165	DVGFINDER
6125/9615	DVGFINDER
6225/704	DVGFINDER
6240/3324	DVGFINDER
6288/7967	DVGFINDER
6504/742	DVGFINDER
6924/2962	DVGFINDER
6940/9114	DVGFINDER
706/6227	DVGFINDER
7242/3859	DVGFINDER
7271/4255	DVGFINDER
745/6507	DVGFINDER
7470/8365	DVGFINDER
7501/8757	DVGFINDER
7574/5084	DVGFINDER
762/8125	DVGFINDER
7710/5359	DVGFINDER
7826/3885	DVGFINDER
7968/6287	DVGFINDER
8076/3193	DVGFINDER
8125/762	DVGFINDER

8224/2747	DVGFINDER
8254/3000	DVGFINDER
8306/7679	DVGFINDER
8357/5016	DVGFINDER
8365/7470	DVGFINDER
8434/7304	DVGFINDER
8581/1436	DVGFINDER
8595/5065	DVGFINDER
8650/9349	DVGFINDER
8665/4479	DVGFINDER
8779/3087	DVGFINDER
8834/921	DVGFINDER
8980/1038	DVGFINDER
9114/6940	DVGFINDER
9177/9776	DVGFINDER
918/8831	DVGFINDER
9640/5891	DVGFINDER
970/3497	DVGFINDER
1004/7321	VIREMA
102/4015	VIREMA
1035/2543	VIREMA
1040/8978	VIREMA
1051/2302	VIREMA
1071/1973	VIREMA
1460/2998	VIREMA
1502/4275	VIREMA
163/4932	VIREMA
1707/7655	VIREMA
176/490	VIREMA
1784/6060	VIREMA
1803/5741	VIREMA
1909/8273	VIREMA
2354/4290	VIREMA
2366/3648	VIREMA
2387/1501	VIREMA
2677/3537	VIREMA
2964/6926	VIREMA
2983/7883	VIREMA
3000/1458	VIREMA
3002/8256	VIREMA
3200/9054	VIREMA
3324/6240	VIREMA
3409/4133	VIREMA

3460/7774	VIREMA
3497/970	VIREMA
3516/5147	VIREMA
3537/2677	VIREMA
3598/3792	VIREMA
3611/4844	VIREMA
3792/3598	VIREMA
3859/7242	VIREMA
3885/7826	VIREMA
4022/7019	VIREMA
4062/2928	VIREMA
4133/3409	VIREMA
4255/7271	VIREMA
4560/4654	VIREMA
4600/5215	VIREMA
4653/4559	VIREMA
4795/4531	VIREMA
4846/3613	VIREMA
490/176	VIREMA
4964/8435	VIREMA
50/6093	VIREMA
5016/8357	VIREMA
5083/7573	VIREMA
5147/3516	VIREMA
5149/8484	VIREMA
55/8684	VIREMA
552/5442	VIREMA
5893/9642	VIREMA
6000/6040	VIREMA
6040/6000	VIREMA
6104/4165	VIREMA
6125/9615	VIREMA
6225/704	VIREMA
6240/3324	VIREMA
6288/7967	VIREMA
6504/742	VIREMA
6924/2962	VIREMA
6940/9114	VIREMA
706/6227	VIREMA
7242/3859	VIREMA
7271/4255	VIREMA
745/6507	VIREMA
7470/8365	VIREMA

7501/8757	VIREMA
7574/5084	VIREMA
762/8125	VIREMA
7710/5359	VIREMA
7968/6287	VIREMA
8076/3193	VIREMA
8125/762	VIREMA
8224/2747	VIREMA
8254/3000	VIREMA
8306/7679	VIREMA
8357/5016	VIREMA
8432/4967	VIREMA
8434/7304	VIREMA
8484/5149	VIREMA
8581/1436	VIREMA
8595/5065	VIREMA
8650/9349	VIREMA
8665/4479	VIREMA
8779/3087	VIREMA
8834/921	VIREMA
8980/1038	VIREMA
9114/6940	VIREMA
9177/9776	VIREMA
918/8831	VIREMA
9347/8648	VIREMA
9640/5891	VIREMA
970/3497	VIREMA
1051_2302	VODKA2
1784_6060	VODKA2
1909_8273	VODKA2
2365_3647	VODKA2
2964_6926	VODKA2
3002_8256	VODKA2
3516_5147	VODKA2
3859_7242	VODKA2
4022_7019	VODKA2
4166_6105	VODKA2
4479_8665	VODKA2
4531_4795	VODKA2
4560_4654	VODKA2
5016_8357	VODKA2
5065_8595	VODKA2
5892_9641	VODKA2

6000_6040	VODKA2
706_6227	VODKA2
743_6505	VODKA2
8649_9348	VODKA2
8649_9348	VODKA2

SUPPLEMENTAL TABLE 2

List of junctions in main text table 3 that are presented as averages of a group of similarly related junctions (column 2), along with what raw junctions went into that average (column 3) and the instance counts of those raw junctions). Note that the instance counts of all the raw junctions are combined to give the instance count of the average junction: for example, for CYMV average junction 152/47, its instance count would be listed as 161, which is the sum of 61, 54, and 46.

Virus	"average" junction listed in main text table 3	Junctions that went into that average	Instance counts of those junctions	Program
CYMV	152/47	152/52, 152/40, 152/48	61, 54, 46	VIREMA
CMV	38/16	38/15, 38/17	5, 5	VIREMA
COVID	29871/29890	29871/29895, 29871/29894, 29871/29893, 29871/29892, 29871/29891, 29871/29890, 29871/29889, 29871/29888, 29871/29887, 29871/29886 29871/29885	2, 2, 2, 2, 2, 2, 2, 2, 2, 2, 2	VIREMA
BMV	786/794	790/797, 788/795, 787/794, 787/795, 786/794, 786/793, 786/796, 786/795, 785/793, 785/795, 785/792, 784/794, 784/792, 783/792, 782/790	12, 12, 105, 26, 125, 113, 19, 16, 51, 33, 16, 13, 12, 10, 33	DI-TECTOR
COVID	60/28250	58/28249, 58/28250, 59/28250, 60/28250, 61/28250, 58/28251, 60/28251, 61/28251	1, 1, 2, 4, 9, 1, 15, 89	DI-TECTOR
CymRSV	691/3024	680/3022, 681/3023,	1, 11, 4, 1, 1, 2, 13, 3, 9, 2	DI-TECTOR

		691/3025, 691/3023, 692/3023, 694/3022, 695/3023, 695/3022, 696/3024, 697/3024		
BMV	765/762	761/759, 762/759, 766/762, 769/767	16, 16, 16, 18	DVGFINDER
CymRSV	695/3024	691/3023, 691/3025, 692/3023, 694/3022, 695/3022, 695/3023, 696/3024, 697/3024	1 FOR ALL	DVGFINDER
TCV	1452/249	1451/249, 1452/249	4 for both	DVGFINDER
TCV	1449/2431	1447/2429, 1451/2433	4 for both	DVGFINDER
TCV	1452/3151	1450/3147, 1454/3154	4 for both	DVGFINDER
TCV	1452/3891	1452/3886, 1451/3895	4 for both	DVGFINDER
TCV	193/3688	193/3686, 193/3689	4 for both	VODKA2
CYMV	5092/5094	5090/5092, 5093/5095	1 for both	DG-SEQ
CYMV	5064/5066	5063/5065, 5065/5067	1 for both	DG-SEQ

SUPPLEMENTAL TABLE 3

List of unrelated junctions tied for "most common" during the investigation into the most frequently reported junction in the program outputs (main text table 3).

Virus	Program	Junction List	Number of hits
TuMV	VIREMA	6924/2962<br>6225/704<br>9347/8648<br>1004/7321<br>4600/5215<br>9640/5891<br>8254/3000<br>7501/8757<br>1803/5741<br>1071/1973<br>3885/7826<br>8357/5016<br>4795/4531<br>6104/4165<br>6040/6000<br>6504/742<br>1502/4275<br>5083/7573<br>3611/4844<br>3598/3792<br>7470/8365<br>8665/4479<br>7242/3859<br>5147/3516<br>4653/4559<br>8595/5065<br>918/8831<br>5149/8484<br>552/5442<br>2983/7883<br>176/490<br>2964/6926<br>8650/9349<br>6000/6040<br>7574/5084<br>8581/1436<br>4846/3613<br>3859/7242	1 (for all)

		4560/4654<br>3002/8256<br>8834/921<br>490/176<br>8779/3087<br>5016/8357<br>2366/3648<br>4022/7019<br>1784/6060<br>55/8684<br>745/6507<br>8076/3193<br>3792/3598<br>4062/2928<br>8224/2747<br>706/6227<br>3516/5147<br>50/6093<br>1909/8273<br>1051/2302<br>5893/9642<br>8484/5149<br>6288/7967<br>970/3497<br>1460/2998<br>762/8125<br>9177/9776<br>3537/2677<br>7710/5359<br>3497/970<br>8980/1038<br>1040/8978<br>8125/762<br>7968/6287<br>3460/7774<br>1707/7655<br>2677/3537<br>163/4932<br>102/4015<br>3000/1458<br>4964/8435<br>3200/9054<br>6940/9114<br>2354/4290	

		2387/1501<br>9114/6940<br>8432/4967<br>1035/2543<br>8434/7304<br>3409/4133<br>4133/3409<br>6240/3324<br>3324/6240<br>6125/9615<br>8306/7679<br>4255/7271<br>7271/4255	
TCV	VIREMA	1177/860<br>3397/3425<br>1509/2281<br>2495/3981<br>3340/1361<br>796/1347<br>3952/233<br>3214/3844<br>2986/1727<br>3425/1340<br>2782/2429<br>2871/658<br>3034/1571<br>1183/3772<br>1073/3110<br>1105/2858<br>2262/726<br>3522/1726<br>2584/3756<br>342/3461<br>1124/970<br>2268/2037<br>374/982<br>2356/3324<br>3324/2693<br>3282/3668<br>1682/737<br>259/3622<br>2581/2373<br>2869/2051<br>501/1496	4 (for all)

		2419/2685<br>2930/2655<br>3276/3493<br>2991/1100<br>2091/3077<br>2687/274<br>3593/1769<br>1249/593<br>2174/3619<br>1216/2091<br>2753/1458<br>1453/32<br>3268/1559<br>1670/3013<br>2090/1862<br>1841/1237<br>194/3570<br>3791/3016<br>3150/2912<br>1118/2219<br>1354/3133<br>3114/505<br>1760/3982<br>419/3437<br>3608/3828<br>380/1726<br>221/2579<br>2021/1195<br>1015/1251<br>2239/659<br>92/457<br>1896/3898<br>1805/2110<br>3844/1351<br>3821/3754<br>1304/176<br>1382/1988<br>2838/3461<br>1634/405<br>3013/2910<br>2654/3122<br>3682/2868<br>3523/554<br>3281/3602	

		745/2471<br>999/3084<br>2583/3384<br>1544/1528<br>563/1125	
TuMV	DVGFINDER	4795/4531<br>5147/3516<br>6040/6000<br>6104/4165<br>6225/704<br>6504/742<br>6924/2962<br>7242/3859<br>8254/3000<br>8357/5016<br>8595/5065<br>8665/4479<br>9640/5891<br>1051/2302<br>1784/6060<br>1909/8273<br>2366/3648<br>2964/6926<br>3002/8256<br>3516/5147<br>3859/7242<br>4022/7019<br>4560/4654<br>5893/9642<br>6000/6040<br>706/6227<br>745/6507<br>8650/9349	1 (for all)
